# The Demographics of Patients With Dermal Filler Complications

**DOI:** 10.1111/jocd.70043

**Published:** 2025-03-04

**Authors:** Saman Al‐Zahawi, Ala Ehsani, Tahmineh Jozdani, Amirhossein Rahimnia, A. H. Ehsani, Zahra Razavi, Seyed Naser Emadi

**Affiliations:** ^1^ Department of Dermatology, Razi Hospital Tehran University of Medical Sciences (TUMS) Tehran Iran; ^2^ School of Medicine Shahid Beheshti University of Medical Science Tehran Iran; ^3^ School of Medicine Tehran University of Medical Science Tehran Iran

**Keywords:** demography, dermal filler reactions, dermal fillers, hyaluronic acid

## 1 Introduction

Over the past two decades, dermal fillers have gained widespread popularity as nonsurgical cosmetic treatments globally, even supplanting surgical procedures in some cases. Common types of dermal fillers include calcium hydroxyapatite, polylactic acid, polymethyl methacrylate, and hyaluronic acid [1].

Hyaluronic acid emerged as the most acceptable filler for facial augmentation and facial contouring because of its suitable price over surgical operation, excellent cosmetic efficacy, well tolerated by the dermal tissue, good safety profile, ease of reversibility by hyaluronidase enzyme if medically or cosmetically unwanted, and lastly few possible side effects [2]. Even though the overall side effects are few and mild, the profound injections of nonstandard dermal fillers in some communities by nonprofessional staff have increased the prevalence of its side effects. Indeed, standard dermal fillers and correct techniques will aid in decreasing the incidence of adverse effects of dermal fillers. These adverse side effects are categorized according to the severity into mild, moderate, and severe, or according to the nature of the involved tissue into ischemic or nonischemic [3]. Some authors prefer to classify dermal filler complications into early and late complications depending on the time of occurrence after the dermal filler injection. Early‐onset complications occur within 24 h, delayed early complications occur within the first 4 weeks, and late complications occur after 4 weeks [4].

Adverse side effects range from mild events such as ecchymosis, erythema, swelling, itching, allergic reactions, and subcutaneous nodular formation to more severe unpleasant, disastrous events like infections (bacterial, herpes simplex, abscess formation), granuloma formation, vascular occlusion, permanent vision loss, and nerve palsies [5]. Fortunately, the last three aforementioned adverse events are infrequent, with a possible underreporting. To ensure the safe and effective use of dermal fillers, it is essential to determine the factors that contribute to complications and minimize their occurrence. Beyond the well‐established factors of filler type and injection technique, patient demographics play a significant role in the development of dermal filler complications [6].

This study analyzed the demographic characteristics of patients who presented with dermal filler complications at Razi Dermatological Hospital, Tehran University of Medical Sciences, during the year 2023.

## 2 Method

In a retrospective, cross‐sectional, descriptive study of patients who visited Razi Dermatological Hospital/Tehran University for Medical Science, for dermal filler complications in 2023, demographic features of patients were obtained through a questionnaire containing data regarding age, sex, type of injected filler, type of reaction, site of reaction, history of smoking, previous history of lupus erythematosus, history of atopic dermatitis, an allergic reaction, and any drug intake. The collected data were analysed by descriptive–inferential statics; moreover, central dispersion indices, average, and standard deviation were used to analyze data.

## 3 Results

Fifty‐four patients with dermal filler complications were included in our study. There were 48 female patients and 6 male patients. The youngest patient was 21 years old, whereas the oldest was 55. The median age was 34.11 years with a standard deviation of 8.37. Dermal filler complications were significantly higher in female patients (88.9%), and this may be attributed to the higher number of females undergoing dermal filler injections. Hyaluronic acid filler was the most common implicated filler, comprising about 87.2%, and vitamin E comprised only 12.8% of the type of injected filler. Dermal filler complications were observed in the following anatomical sites: nasolabial fold 37%, nose 22.2%, chin 14.8%, lip 11.1%, glabellar complex 5.6%, cheek 5.6%, and multiple anatomical site injection in 3.7%. Our study showed that 18.5% of the patients with dermal filler complications were smokers and 27.8% were vape smokers. Regarding associated dermatological disease among patients with dermal filler complications, allergic contact dermatitis was the most common at 16.7%, followed by psoriasis and atopic dermatitis.

**FIGURE 1:**
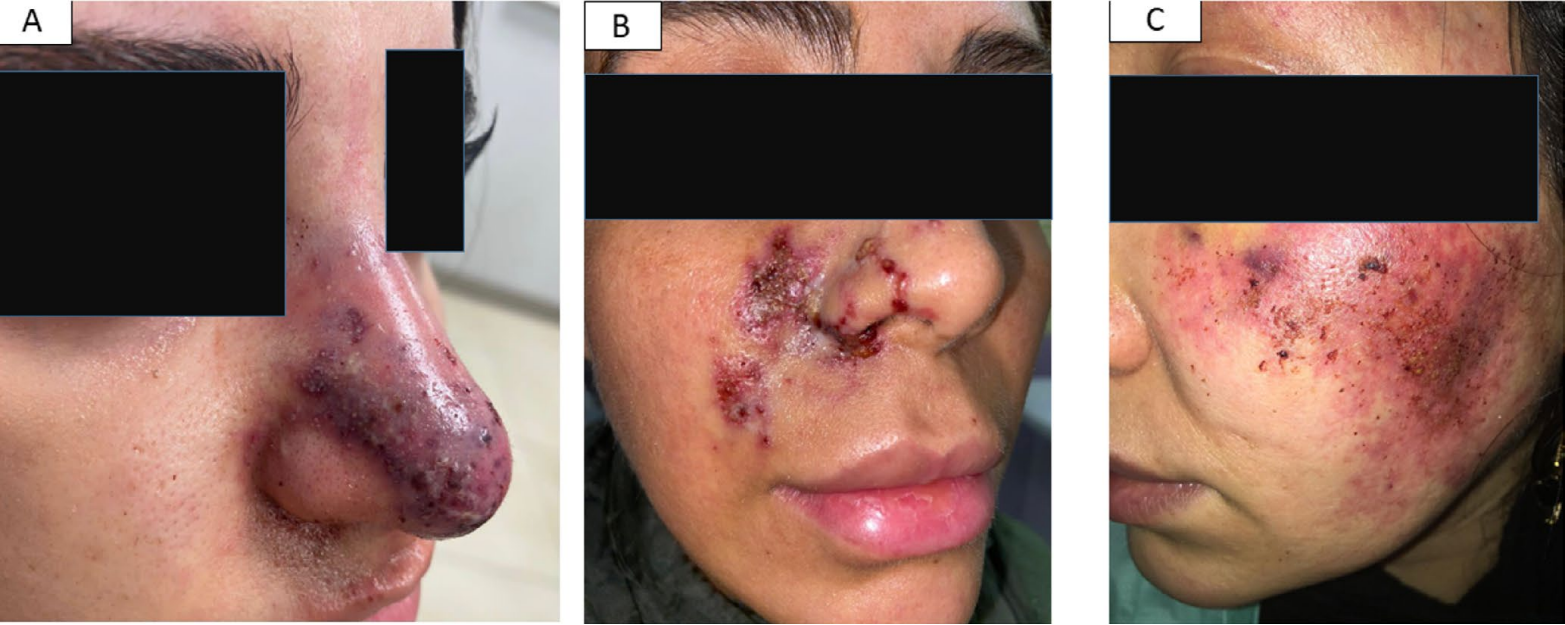
Early vascular necrosis in nose (A), nasolabial fold (B), and cheek (C). (photo by Dr. Seyed Naser Emadi, Tehran, Iran).

**FIGURE 2:**
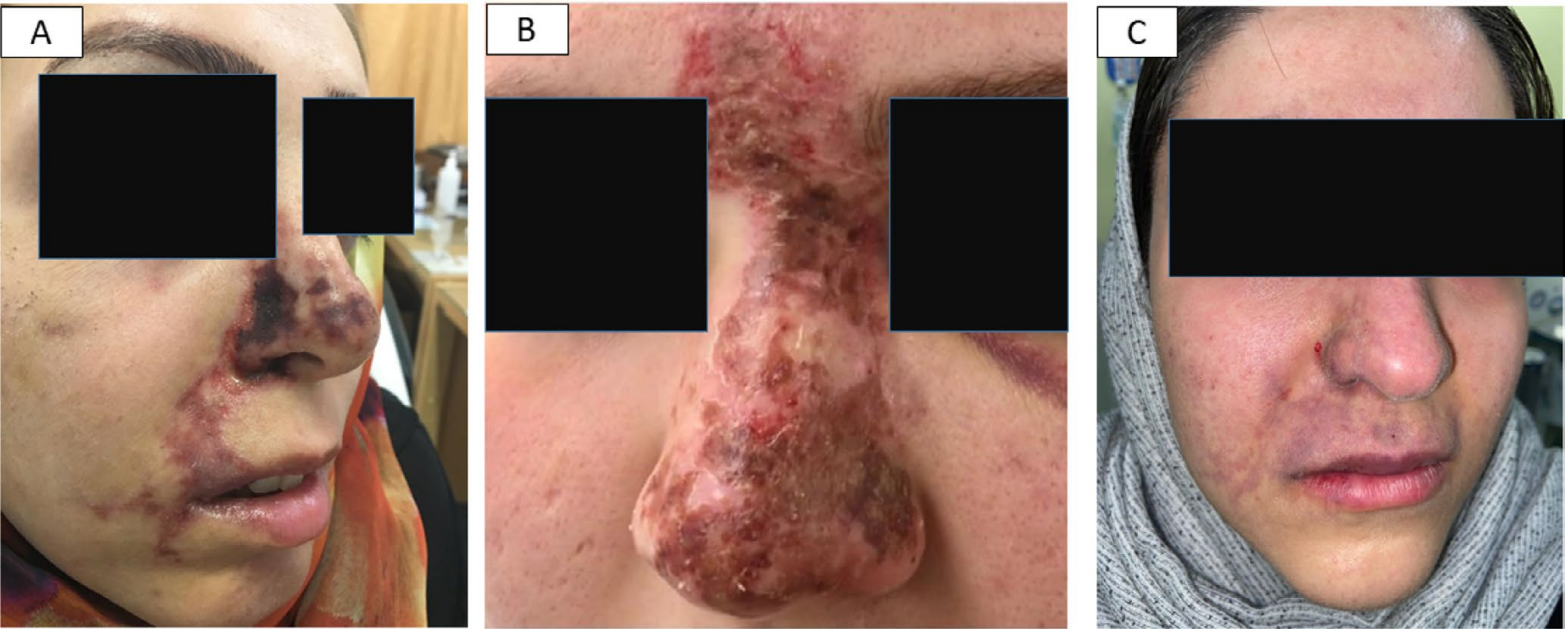
Multiple sites vascular necrosis in nose and nasolabial fold (A), nose and glabella (B), and nasolabial fold and upper lip (C). (photo by Dr. Seyed Naser Emadi, Tehran, Iran).

**FIGURE 3:**
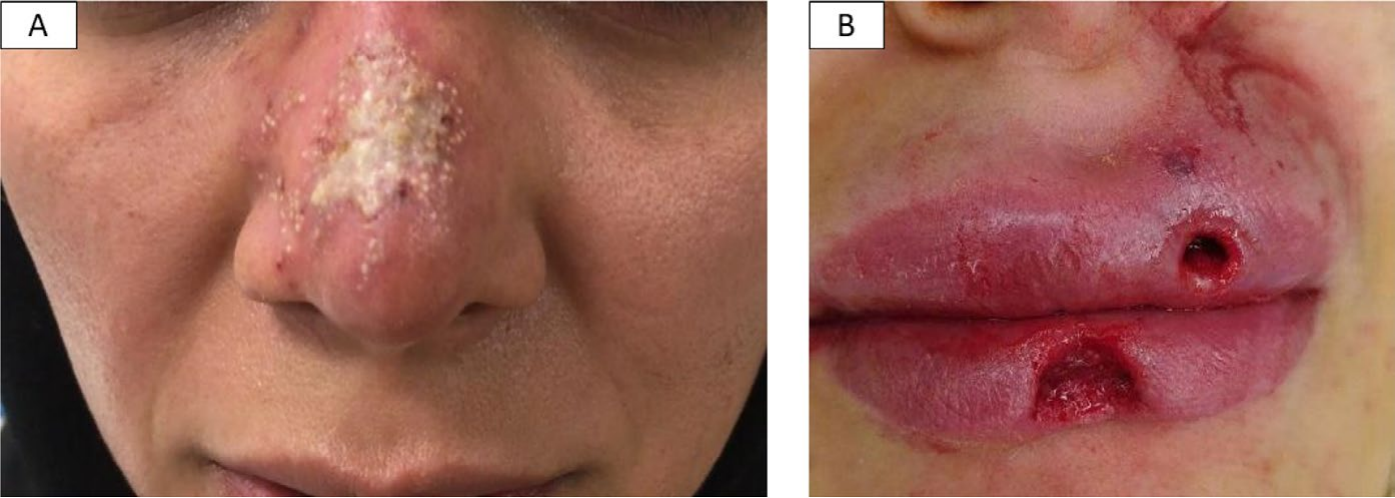
Infection following dermal filler complication, herpes simplex reactivation (A), and ulceration with discharge (B). (photo by Dr. Seyed Naser Emadi, Tehran, Iran).

**FIGURE 4:**
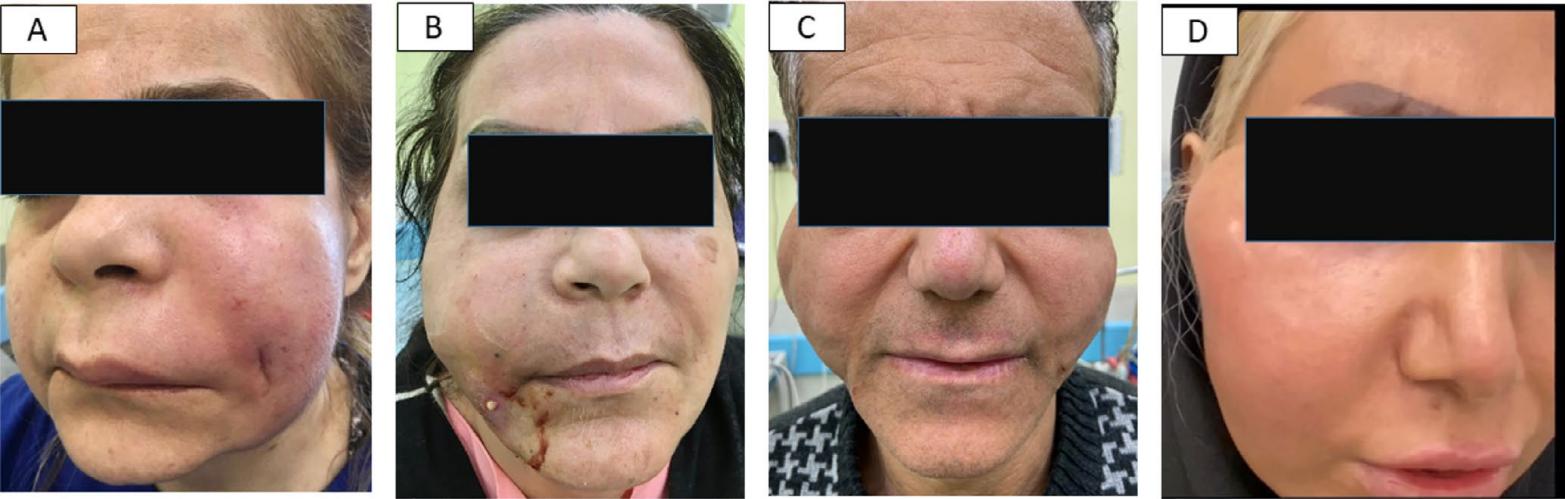
Late complications (years after injecting vitamin E) presenting with recurrent lumpiness and erythema resembling eryseplas (A), recurrent abscess formation with white discharge (B), bilateral lumpiness (C), and unilateral cheek lumpiness (D). (photo by Dr. Seyed Naser Emadi, Tehran, Iran).

Ischemic complications were seen in 51.9% of patients (Figures 1 and 2) versus nonischemic changes in 48.1% (Figures 3 and 4).

Although 33.3% of patients had previous dermal filler injections, only 1.9% of patients were having previous history of dermal filler complications of the nasolabial fold. General physicians accounted for 46.3% of the provided dermal filler injectors, and nonmedical staff accounted for 46.3% of the dermal filler injectors. Only 7.4% of the dermal filler injection complications were conducted by certified physicians.

A statistically significant association was observed between the type of injected filler and dermal filler complications (*p* value < 0.05). All vitamin E filler injection complications were nonischemic, whereas hyaluronic acid injection caused equally ischemic and nonischemic complications (15 cases of vascular complication versus 19 nonvascular complications).

A significant statistical association was observed between anatomical site injection and dermal filler complication (*p* value < 0.05). Ischemic changes were observed mostly after dermal filler injection of the nasolabial fold and the nose, whereas nonischemic changes were seen in the nasolabial fold, lip, and chins. Also, a significant statistical association was observed between smoking and type of dermal filler complications (*p* value < 0.05), in which the incidence of ischemic dermal filler complications was higher in those who smoke and less common incidence of ischemic dermal filler complications in those who did not smoke. No association between dermal filler complications with age, sex, lupus, and other underlying dermatological diseases was observed.

## 4 Discussion

Dermal fillers have gained significant popularity in cosmetics in recent years due to their generally favorable safety profile. However, maintaining this high safety record hinges on the meticulous use of proper injection techniques and the employment of approved high‐quality dermal fillers. In addition, individual demographic factors should be assessed thoroughly for a possible complication related to changes in vascularity of the region by a previous surgical operation, increased chance of ecchymosis in patients with anticoagulant medications, high chance of vascular complication in specific anatomic sites, and possible vascular necrosis in smokers with change of blood vessel viscosity and decreased skin vascularity.

Although hyaluronic acid is a well‐known dermal filler for its safety profile, it is also known to be the most dermal filler responsible for vascular necrosis and even irreversible blindness in some cases due to ophthalmic artery occlusion [7]. Utmost awareness should be taken into account when injecting such type of dermal filler into a specific anatomic site, such as the nose or glabella, as significant vascular necrosis has been reported [8, 9]. In our study, the nasolabial fold was the most frequent site of vascular compromise, aligning with previous findings that identified this area as a high‐risk zone [10].

Smoking is also considered to be an important demographic factor in increasing the risk of dermal filler complications, as previous reports showed a decrease in vascularity of the skin and skin flap failure in active smoker patients undergoing skin flap [11]. The same rule of decreased vascularity of the skin may be applied for vascular necrosis after dermal filler injection in active smoker patients seen in our study; however, the role of active smoking as a risk factor for vascular necrosis after dermal filler injection is debatable as the majority of patients with vascular necrosis have no history of smoking [12]. Our study revealed that the occurrence of dermal filler complications has no association with age, sex, or specific dermatological conditions.

The approach to managing complications in this study was determined by a combination of factors, including the ischemic or nonischemic nature of the complication, the temporal relationship to filler injection, the specific type of dermal filler used, and the presence of collections or foreign body granulomas as identified by ultrasound.

For instance, the patient illustrated in Figure 1A presented with early vascular complications after receiving hyaluronic acid injections in the nose. Following our department's protocol for ischemic complications, she underwent treatment with four consecutive 750‐unit doses of hyaluronidase enzyme administered every 2 h, along with antibiotics, antivirals, and aspirin. Unfortunately, this patient was lost to follow‐up, hindering the assessment of the treatment's effectiveness. The higher dose of hyaluronidase for early vascular complications was determined based on the extent of the necrotic area and supported by findings from previous research [13].

Patients depicted in Figure 4A–D, who received vitamin E injections, experienced complications months or even years after their initial dermal filler procedures. In the latter group, an initial ultrasound evaluation was performed on the swollen area. Patients with collections or abscesses were treated with drainage followed by prolonged antibiotic and immunosuppressive therapy. Those exhibiting signs of granuloma formation underwent multiple treatment regimens, including methotrexate, prednisolone, tofacitinib, minocycline, doxycycline, intralesional corticosteroids, and intralesional hyaluronidase injections. However, the outcomes of these treatments were inconsistent and often disappointing in the short‐term follow‐up period.

This study has several limitations, including its retrospective design, small sample size, data limitations, the potential influence of confounding factors such as filler brand, and the lack of long‐term follow‐up. These limitations may weaken the strength of the conclusions. To strengthen future studies regarding the demographics of filler complications, we recommend a larger, multicenter study that meticulously identifies and addresses potential confounding factors.

## 5 Conclusion

Our study showed that the type of dermal filler complications (ischemic versus nonischemic) has an association with smoking, anatomical site injection, and type of dermal filler injection. The high incidence of ischemic dermal filler complication in the nasolabial fold and nose could be explained by the high rate of dermal filler injection in the nasolabial fold, nonstandard local filler, and the unexperienced hand, improper technique with poor knowledge of the highly vascularized nasal region. Minimizing the risk of ischemic complications associated with dermal fillers requires a profound understanding of facial anatomy and the meticulous execution of precise injection techniques.

## Author Contributions

Saman Al‐Zahawi, contributing the design of the work, writing and revising the draft, and agreed on all aspects of the study. Ala Ehsani, collecting the data, analyzing the data and agreed on all aspects of the study. Tahmineh Jozdani, collecting the data, analyzing the data and agreed on all aspects of the study. Amirhossein Rahimnia, collecting the data, follow‐up of patients and agreed on all aspects of the study. A.H. Ehsani, supervising the study, analyzing the data, approval for the final manuscript and agreed on all aspects of the study. Zahra Razavi, interpretation of the data, discussion, approval of the final draft and agreed on all aspects of the study. Seyed Naser Emadi, conceptualization, writing and revising the draft, approval of the final draft, and agreed on all aspects of the study.

## Consent

Written informed consent was obtained from the patient's parents.

## Conflicts of Interest

The authors declare no conflicts of interest.

## Data Availability

The data that support the findings of this study are not publicly available due to privacy reasons but are available from the corresponding author upon request.
